# Quantitative PET imaging of the CD4 pool in nonhuman primates

**DOI:** 10.1007/s00259-022-05940-4

**Published:** 2022-08-27

**Authors:** Insook Kim, Sharat Srinivasula, Paula DeGrange, Brad Long, Hyukjin Jang, Jorge A. Carrasquillo, H. Clifford Lane, Michele Di Mascio

**Affiliations:** 1 AIDS Imaging Research Section, Applied/Developmental Research Directorate, Frederick National Laboratory for Cancer Research, Frederick, MD 21702 USA; 2 AIDS Imaging Research Section, Clinical Monitoring Research Program Directorate, Frederick National Laboratory for Cancer Research, Frederick, MD 21702 USA; 3 AIDS Imaging Research Section, Integrated Research Facility, NIAID, NIH, Frederick, MD 21702 USA; 4 Molecular Imaging and Therapy Service, Radiology Department, Memorial Sloan Kettering Cancer Center, New York, NY 10065 USA; 5 Molecular Imaging Branch, Center for Cancer Research, NCI, NIH, Bethesda, MD 20892 USA; 6 Laboratory of Immunoregulation, Division of Intramural Research, NIAID, NIH, Bethesda, MD 20892 USA; 7 AIDS Imaging Research Section, Division of Clinical Research, NIAID, NIH, Bethesda, MD 20892 USA

**Keywords:** Immuno-PET, CD4R1 antibody, Ibalizumab, SIV-infected, Gut CD4 pool, Binding potential

## Abstract

**Purpose:**

Previous SPECT and PET semi-quantitative in vivo imaging studies in monkeys have demonstrated specific uptake of radiolabeled rhesus recombinant anti-CD4 monoclonal antibody fragment CD4R1-F(ab΄)_2_ in the spleen and clusters of lymph nodes (LNs) but yielded conflicting results of imaging the gut CD4 + T-cell pool. Here, using PET dynamic imaging with kinetic analysis, we performed a fully quantitative CD4 imaging in rhesus macaques.

**Methods:**

The biodistributions of [^89^Zr]Zr-CD4R1-F(ab΄)_2_ and/or of [^89^Zr]Zr-ibalizumab were performed with static PET scans up to 144 h (6 days) post-injection in 18 rhesus macaques with peripheral blood CD4 + T cells/μl ranging from ~ 20 to 2400. Fully quantitative analysis with a 4-h dynamic scan, arterial sampling, metabolite evaluation, and model fitting was performed in three immunocompetent monkeys to estimate the binding potential of CD4 receptors in the LNs, spleen, and gut.

**Results:**

The biodistributions of [^89^Zr]Zr-CD4R1-F(ab΄)_2_ and [^89^Zr]Zr-ibalizumab were similar in lymphoid tissues with a clear delineation of the CD4 pool in the LNs and spleen and a significant difference in lymphoid tissue uptake between immunocompetent and immunocompromised macaques. Consistent with our previous SPECT imaging of [^99m^Tc]Tc-CD4R1-F(ab΄)_2_, the [^89^Zr]Zr-CD4R1-F(ab΄)_2_ and [^89^Zr]Zr-Ibalizumab uptakes in the gut were low and not different between uninfected and SIV-infected CD4-depleted monkeys. Ex vivo studies of large and small intestines confirmed the in vivo images.

**Conclusion:**

The majority of specific binding to CD4 + tissue was localized to LNs and spleen with minimal uptake in the gut. Binding potential derived from fully quantitative studies revealed that the contribution of the gut is lower than the spleen’s contribution to the total body CD4 pool.

**Supplementary Information:**

The online version contains supplementary material available at 10.1007/s00259-022-05940-4.

## Introduction

Studies published between 1995 and 2005 [1–5] that underscored the importance of the insult induced by HIV-1 in humans and SIV in rhesus macaques on the gut for disease pathogenesis primarily referenced the same three studies [6–8] to claim the gut harbors the majority of T lymphocytes. Though the definition of gut varied and the information on the total number of immune cells was lacking in these early reports, it became a widespread belief among immunologists and HIV researchers that most lymphocytes, and consequently most CD4 + T-cells, the primary target cell of HIV/SIV infection, reside in the gut.

In the past two decades, SPECT in vivo studies from our team [9–11] have generated the first images of the whole-body CD4 pool in rhesus macaques and also shown the capability of this novel imaging system to visualize changes in true repopulation of CD4 + cells in tissues versus changes in the trafficking of CD4 + cells and their relationships with changes in peripheral blood (PB) CD4 + cells. This is particularly useful, for instance, in studying the dynamics of CD4 + cell repopulation in the PB following perturbation of lymphocyte homing, e.g., through a hematopoietic stem cells mobilizing agent (AMD3100), as we have shown a few years ago using [^99m^Tc]Tc-CD4R1-F(ab΄)_2_ mAb probe [10]. Our CD4-pool SPECT nonhuman primates (NHP) in vivo studies were followed by CD4-pool PET NHP in vivo studies by another team that adopted this imaging system using [^64^Cu]Cu-CD4R1-F(ab΄)_2_ to study the efficacy of an immunotherapeutic intervention antagonizing lymphocyte homing into the gut [12, 13]. Their studies showed the feasibility of imaging the CD4-pool in the clusters of lymph nodes, the spleen, as well as in the gastrointestinal tract (colon and small bowel) which, based on their image interpretation, appears to be an anatomic compartment very rich in CD4 cells, as recently reviewed [14, 15].

A review published in 2007 by Ganusov and de Boer [16] questioned the notion of the gut being the major compartment of lymphocytes utilizing quantitative data on immune cell numbers estimated from tissue extraction coupled with tissue staining techniques from various anatomic compartments of different mammalian species. They estimated that only approximately 11% and 15% of the total body lymphocytes are located in the gut and the spleen respectively, and concluded that the spleen and LNs are the major immune compartments in humans. Our previous CD4-pool in vivo imaging studies in nonhuman primates also showed the feasibility of imaging the CD4 pool in LNs and the spleen; however, in contrast with the CD4-pool PET studies mentioned above [12, 13], the radiotracer uptake in the gut was found to be very low and not different between healthy and SIV-infected animals. Using that data, we concluded that the gut CD4 pool is lower than the spleen CD4 pool alone. While both groups used the same primatized OKT4A monoclonal antibody (mAb) (CD4R1) and observed similar levels of anti-CD4 probe uptake in the clusters of LNs and the spleen of healthy animals as well as similar reductions in SIV-infected animals, the interpretation of the gut uptake from the images was significantly different as discussed elsewhere [11, 17]. Though the experimental settings were different among the studies (radiolabel, camera technology, mAb mass, and biodistribution time), the conclusions from both groups relied upon the semi-quantitative approach of standardized uptake value (SUV) to infer organ-specific CD4 receptor concentrations, an indirect measurement of the number of CD4 + cells per unit tissue volume.

To overcome some of the recognized pitfalls of semi-quantitative image analysis [18], in the present study, we utilized dynamic PET imaging with kinetic analysis methods coupled with ex vivo validation analysis and calculated a quantitative measure of tissue receptor density to address the discrepancy in gut uptakes and provide additional data on the contribution of the gut to the CD4 + T-cell pool. To corroborate our whole-body in vivo findings with CD4R1-F(ab′)_2_ mAb (clone OKT4A) that binds to an epitope on domain 1 of the CD4 receptor [19], we also imaged the CD4 pool with ibalizumab, a CD4 domain 2 directed humanized mAb that binds to the interface between domain 1 and domain 2 of CD4 [20] by radiolabeling both anti-CD4 probes with positron-emitting ^89^Zr (half-life 78.4 h) to extend the biodistribution time up to 6 days (144 h) post-injection.

## Materials and methods

### Antibodies

Rhesus recombinant antibody, CD4R1-OKT4A/rhIgG1(CD4R1), was obtained from NIH Nonhuman Primate Reagent Resource and was produced by grafting onto a rhesus scaffold using rhesus germline variable region in complementarity-determining regions representing the anti-CD4 antibody OKT4A [21].

Ibalizumab is a recombinant humanized anti-CD4 IgG4 mAb approved in the USA and Europe by the FDA and EMEA, respectively as TROGARZO™ (Ibalizumab-uiyk Injection, Theratechnologies Inc.) for use as part of a combination antiretroviral regimen in heavily treatment-experienced patients with multidrug-resistant HIV-1 infection failing their current antiretroviral regimen [22]. A buffer exchange of ibalizumab vial content was performed with PBS 1X (pH 7.2) using a Zeba Spin Desalting column (7 K MWCO, Pierce Biotechnology, Rockford, Illinois) before the conjugation with desferrioxamine (Df) (see Supplemental Materials and Methods).

### Radiolabeling

[^89^Zr]Zr-Df-CD4R1-F(ab′)_2_ ([^89^Zr]CD4R1-F(ab′)_2_) and [^89^Zr]Zr-Df-ibalizumab intact ([^89^Zr]ibalizumab) were prepared as previously described, but with minor modifications [23]. Three hundred seventy megabecquerel of ^89^Zr in 1 M oxalic acid was neutralized with 2 M sodium bicarbonate, followed by the addition of 0.5 M HEPES buffer (200 μL, pH 7.1–7.3) and gentisic acid (5 mg/mL, 25μL, pH 6.5). Df-conjugated mAb (approximately 0.4 mg or 3 mg of Df-F(ab′)_2_-CD4R1 for high (100 μg) and low specific activity (1000 μg) doses, respectively) was added, and the pH was readjusted to 7.3–7.5 with 0.1 M Na_2_CO_3_. The reaction vial was incubated for 1 h at 30 °C. The labeled product was purified with a PD-10 column eluted with PBS 1 × (pH 7.2). The PD-10 column was pretreated with 25 mg HSA and 1 μmol DTPA to block non-specific protein binding sites. The radiochemical purity was assessed by analytical size exclusion HPLC (Gilson, Middleton, WI, USA) equipped with a TSK gel G4000PWxL column (7.8 × 300 mm, 10 μm, TOSOH Bioscience; 0.067 M sodium phosphate/0.15 M sodium chloride with 0.1 M KCl, pH 6.8; 1.0 mL/min) equipped with an on-line flow radioactivity detector (Bioscan Inc., Washington, DC, USA). The radiolabeling yield was determined based on the distribution of ^89^Zr between ^89^Zr labeled antibodies (retention time: 9.2 min) and unbound ^89^Zr (retention time: 10.7 min) on the HPLC profiles obtained before the purification. Radiochemical purity greater than 90% was utilized for both in vitro and in vivo imaging studies.

### Animals

Eighteen Indian rhesus macaques (*Macaca mulatta*) aged 3–14 years were used in this study. The animal characteristics are shown in Table S1. Of the eighteen macaques, fifteen received either [^89^Zr]CD4R1-F(ab′)_2_ or [^89^Zr]ibalizumab mAb, and three macaques received both radiolabeled mAbs. The seven SIVmac239-nef-stop infected macaques were previously used in an anti-α_4_β_7_ mAb study [24]. However, at the time of PET imaging, they were OFF antiretroviral therapy (ART). The SIVmac239 and SIVmac251 chronically infected animals were infected for over 1 year (range 14–17 months) and ART naïve. All animal studies were performed under an approved National Institute of Allergy and Infectious Diseases (NIAID) Animal Care and Use Committee (ACUC) (Bethesda, Maryland, USA) protocol. Animals were cared for in accordance with the Guide for the Care and Use of Laboratory Animals [25] and were housed in a biosafety level 2 facility, with biosafety level 3 practices followed.

### Study design

As shown in the study outline (Fig. S1), the ten macaques used for PET imaging of [^89^Zr]CD4R1-F(ab′)_2_ were divided into two groups to assess differences in biodistribution between ~ 100 μg versus ~ 1000 μg of [^89^Zr]CD4R1-F(ab′)_2_. The 100-μg dose group animals (four uninfected and two SIV infected) received (mean ± SD) 110 ± 15 μg and 68.8 ± 11.2 MBq of [^89^Zr]Zr-CD4R1-F(ab΄)_2_, and static PET scans were performed in 5 of 6 monkeys at multiple time points post-radioligand injection (p.r.i). For estimation of binding potential, a 4-h dynamic scan was performed in three uninfected monkeys (DGT7, DFB8, and DG4F). The 1000-μg dose group animals (three uninfected and one SIV infected) received 865 ± 40 μg and 120.8 ± 7.1 MBq of [^89^Zr]Zr-CD4R1-F(ab΄)_2_, and a single static PET scan was performed at ~ 40 h p.r.i.

The eleven macaques used for PET imaging of [^89^Zr]ibalizumab were also divided into two groups (~ 100 μg versus ~ 1000 μg of [^89^Zr]ibalizumab). The 100-μg dose group animals (one uninfected and one SIV infected) received 125 ± 6 μg and 37.4 ± 1.5 MBq of [^89^Zr]ibalizumab. Eight of the nine 1000-μg dose group animals (four immunocompetent and four immunocompromised) received 955 ± 48 μg and 71.5 ± 8.6 MBq of [^89^Zr]ibalizumab. To demonstrate radiotracer specificity in vivo, the ninth 1000-μg dose group animal (uninfected) was co-injected with 5 mg/kg of unlabeled ibalizumab. All eleven animals were imaged with static PET scans at 40–48 h and 134–144 h p.r.i. Four of five immunocompromised SIV-infected macaques (OR8, OR1, DFJT, and DG4W) received C207, an anti-CD3 immunotoxin, on two consecutive days (total 4 doses; each dose 50 μg/kg) 5–7 days before [^89^Zr]ibalizumab injection to further deplete CD4 + T-cells [26].

### Kinetic analysis

**Fig. 1:**
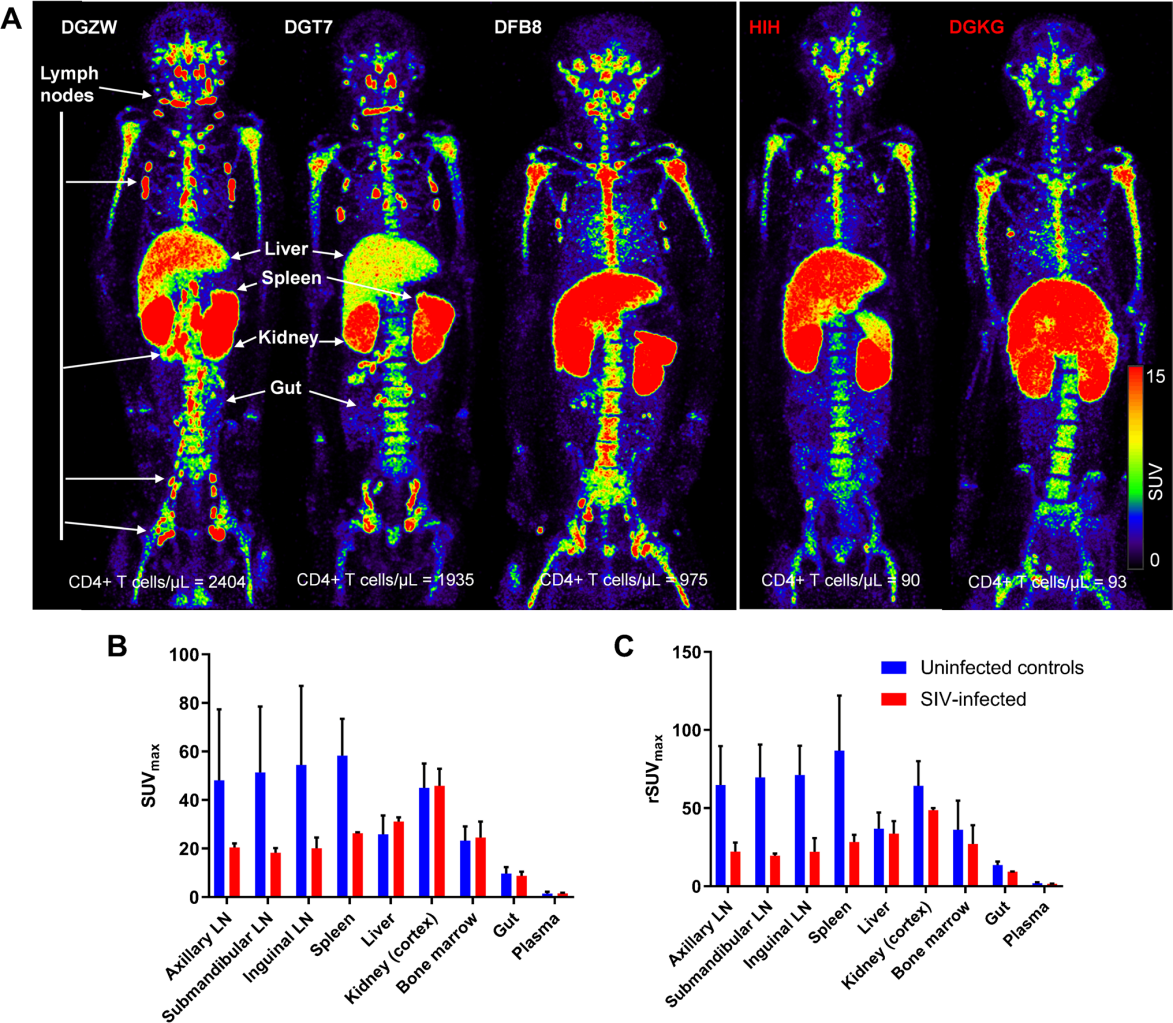
[^89^Zr]CD4R1-F(ab′)_2_ biodistribution following administration of 100 μg mass of mAb. **A** Maximum intensity projection in vivo PET images of rhesus macaques showing the comparison of radioligand uptake between uninfected controls (DGZW, DGT7, and DFB8) and SIV-infected animals (HIH and DGKG) scanned at 40–48 h post-injection. Tissue uptakes were converted to RAINBOW color map as shown in the color bar, where red color indicates the high standardized uptake value (SUV). Comparison of maximum SUV (SUVmax) in tissues and SUV in the plasma (**B**), and blood pool adjusted SUVmax (rSUVmax) (**C**) in tissues and plasma between uninfected controls (blue, *n* = 3) and SIV-infected animals (red, *n* = 2) at 40–48 h post-injection. Plasma SUV was calculated from the gamma counter. Plots are mean values and error bars are standard deviation

For the estimation of binding potential in axillary LN, dynamic scanning was performed at one field-of-view (FOV) for the first 4 h after injection and static imaging at 10 h, 24 h, and 48 h p.r.i in one healthy uninfected control (animal ID: DGT7). For the spleen, 4-h dynamic scanning at 1-FOV followed by static imaging at 48 h and 144 h p.r.i was performed in one healthy uninfected control (DFB8). For the gut, the gut uptake in one healthy uninfected control from a 4-h 3-FOV dynamic scan (DG4F; Suppl Video 2) was combined with static scans of DGT7 (at 10 h and 24 h) and DFB8 (at 48 h and 144 h) to generate the gut time-activity curve for model fitting, justified by low standard deviations of radiotracer uptakes in the gut region as visualized in Fig. 1. The 4-h 1-FOV dynamic PET data had 89 frames with increasing frame duration as follows (# of frames × frame duration): 12 × 10 s, 16 × 30 s, 20 × 1 m, 20 × 3 m, 12 × 5 m, and 9 × 10 m, and the 4-h 3-FOV dynamic PET data had 26 frames as follows: 6 × 30 s, 6 × 1 m, 7 × 3 m, 4 × 5 m, and 3 × 10 m.

Femoral arterial samples were obtained during dynamic scanning of DGT7 and DFB8 and compared to the whole blood (WB) activity derived from a volume of interest (VOI) placed over the cardiac blood pool. The μCi/mL of the WB from the gamma counter overlapped well with the image-derived WB activity as depicted visually in Fig. S9. The area under the curve (AUC) ratio of plasma to WB activity obtained from blood draws was used to calculate the image-derived plasma activity by multiplying this AUC ratio with the VOI image-derived WB activity (C_WB_). HPLC analysis revealed no formation of metabolites or radiolabel disassociation in plasma (Fig. S2B-C). Hence, the metabolite-corrected input function (C_P_) was equated to image-derived plasma activity. The “mean” SUV in the VOI of the PET image was used to determine the tissue concentration of the time-activity curve.

A two-tissue compartment ligand-receptor model previously described [27], and its equation set of 5 parameters (K_1_, k_2_, k_3_, k_4_, and V_B_) was used to best fit the PET image data (Fig. 5A). Best fitting was carried out using PMOD kinetic modeling tool (PMOD Technologies LLC, Switzerland). To give more weight to the latter data points (which were obtained with longer frame duration) compared to the earlier data points (obtained with shorter frame duration), residual weighting was based on frame duration. An iterative Levenberg–Marquardt algorithm was selected for the fitting method, which accumulates information on the covariance matrix and thus enables the calculation of the standard error of the fitted parameters. The image-derived whole-blood (C_WB_) and metabolite-corrected plasma (C_P_) activity data were interpolated with best fitting a 3-exponential decay model. The regional blood volume (V_B_) values of 0.05 and 0.34 were fixed for axillary LN and spleen, respectively [28]. The transfer rate of the ligand from specific binding sites (k_4_) could be assumed equal among the tissues. Hence, coupling k_4_ among the studies, 11 parameters (K_1_, k_2_, and k_3_, for each of 3 tissues (spleen, axillary LN, and the gut), V_B_ for the gut, and a common k_4_) were estimated at once by simultaneously fitting the time-activity data from the spleen, axillary LN, and the gut. To avoid local minima, 1000 random fits were performed with randomized sets of initial parameters to obtain the best-fit parameters resulting in a global minimal chi-square. A V_B_ of 0.071 (95% confidence interval: 0.065–0.078) was estimated from the best fit for the gut, and a common rate constant of 1.76 × 10^−5^ min^−1^ (95% confidence interval: 1.48–2.04 × 10^−5^ min^−1^) was estimated for k_4_.

### Statistical analysis

Unpaired samples were compared with the non-parametric Wilcoxon rank-sum test and the relationship between variables in the cross-sectional analysis was assessed with Spearman rank correlation using Winstat software (Cambridge, MA). A *P*-value < 0.05 was considered statistically significant.

### Ex vivo imaging

Following PET imaging, one uninfected and one SIV-infected from each of the 100 μg and 1000 μg mAb dose groups of [^89^Zr]CD4R1-F(ab΄)_2_ were euthanized at 145–147 h and 42–45 h p.r.i, respectively, and organs were harvested. Individual LNs (axillary, inguinal, submandibular, mesenteric, and retroperitoneal), and small aliquots of the spleen, liver, kidney (cortex), and gut (duodenum, jejunum, ileum, and colon) were weighed, measured in a gamma counter, and decay-corrected radioactivity concentration was calculated. Additionally, in the 1000 μg mAb dose group of [^89^Zr]CD4R1-F(ab΄)_2_, after removing the feces, large sections of the duodenum, jejunum, ileum, and colon, representing ~ 50% (based on weight) of the entire small and large intestines, were placed on a tray and PET imaged.

For the preparation of F(ab′)_2_, conjugation of p-SCN-Df to F(ab´)_2_-CD4R1 and intact Ibalizumab, immunoreactivity binding assays, plasma assay and radio-HPLC, PET/CT imaging, and data analysis, refer to Supplementary Materials and Methods.

## Results

To overcome the limitations of spatial resolution and quantitation of SPECT cameras, we imaged the CD4 pool of rhesus macaques with MultiScan LFER 150 PET/CT (Mediso Medical Imaging Systems, Budapest, Hungary). With smaller PET rings, crystal (and pixel) size, and bore dimension, the novel PET/CT camera specifically designed for nonhuman primates provided a slightly higher spatial resolution and comparable sensitivity to clinical PET cameras [29].

### PET imaging with [^89^Zr]CD4R1-F(ab′)_2_

The specific binding of ^89^Zr radiolabeled CD4R1-F(ab*′*)_2_ to CD4 receptors was tested in vitro using MT4 cells as previously described [11] (Fig. S2A). Before radioligand administration, animals were tested using both plasma binding assay and size exclusion radio-HPLC analysis, and they revealed no sign of pre-existing immunogenicity against CD4R1. Fig. S1 illustrates the imaging schema. Animals were divided into two dose groups: 100 μg mAb dose (high specific activity) and 1000 μg mAb dose groups (low specific activity). The specific activities of the administered [^89^Zr]CD4R1-F(ab΄)_2_ were 0.64 ± 0.14 MBq/μg and 0.14 ± 0.01 MBq/μg for 100 μg (*n* = 6) and 1000 μg (*n* = 4) doses, respectively. The in vivo stability of the radiotracer was tested using size exclusion radio-HPLC analysis of plasma obtained at 10 h and 16 h p.r.i. Greater than 90% of the radioactivity in the plasma eluted with a retention time (9.2 min) identical to that of [^89^Zr]CD4R1-F(ab΄)_2_, suggesting that the imaging probe was stable in vivo (Fig. S2B-C).

#### PET images with high specific activity of [^89^Zr]CD4R1-F(ab΄)_2_

We first compared the PET images using ~ 100 μg of [^89^Zr]CD4R1-F(ab΄)_2_ at 40 h p.r.i with the SPECT images from our earlier studies with ~ 100 μg of [^99m^Tc]Tc-CD4R1-F(ab΄)_2_ obtained at 21 h p.r.i (Fig. S3). Although the SPECT images were not as visually sharp as PET due to less spatial resolution and sensitivity, both PET and SPECT images displayed high levels of probe uptake in the spleen and clusters of axillary, cervical, and inguinal LNs, with clear differences in radiotracer uptake in these tissues between uninfected and SIV-infected, CD4-depleted animals.

Five of six monkeys that received 100 μg mAb were PET imaged at 40–48 h p.r.i (the sixth animal underwent only a 4-h dynamic scan) and semi-quantitative measurements of radiotracer uptake in tissues were obtained (see Supplementary Methods). The 3 uninfected controls (CD4^+^ T-cell counts: 1771 ± 728/μL (mean ± SD)) exhibited 2–threefold higher SUVmax, rSUVmax, and SUVmean in the spleen, and clusters of axillary, cervical, and inguinal LNs compared to the 2 SIV-infected (CD4^+^ T-cell counts: 92 ± 2/μL) (Fig. 1, S4A, and Suppl. Movie 1). Levels of [^89^Zr]CD4R1-F(ab΄)_2_ uptake in the liver, kidney (localized to cortex), and bone marrow were similar between uninfected controls and SIV-infected animals, suggesting the uptake in these organs was the result of non-specific uptake. This semi-quantitative analysis of static PET images was consistent with our previous static SPECT imaging study [11]. The gut uptake was low and similar between uninfected controls and CD4-depleted animals.

To explore if additional biodistribution time resulted in increased gut uptake of [^89^Zr]CD4R1-F(ab΄)_2_, we also imaged two uninfected controls and two SIV-infected monkeys at 137–144 h p.r.i. In these animals, we observed no advantage of imaging at 144 h p.r.i compared to imaging at 48 p.r.i. (Fig. S4C, D). Even at 144 h p.r.i, the gut uptake was low and similar between uninfected and SIV-infected animals. One uninfected control (DGT7) was imaged at two additional time points (10 h and 24 h) earlier than 48 h p.r.i (Fig S5A). Both qualitatively and semi-quantitatively, we observed no significant advantage of imaging later than 10 h since max and mean SUV measures of all tissues of interest had a minor increase in the hours following (Fig. S5B, C).

#### PET images with low specific activity of [^89^Zr]CD4R1-F(ab΄)_2_

**Fig. 2:**
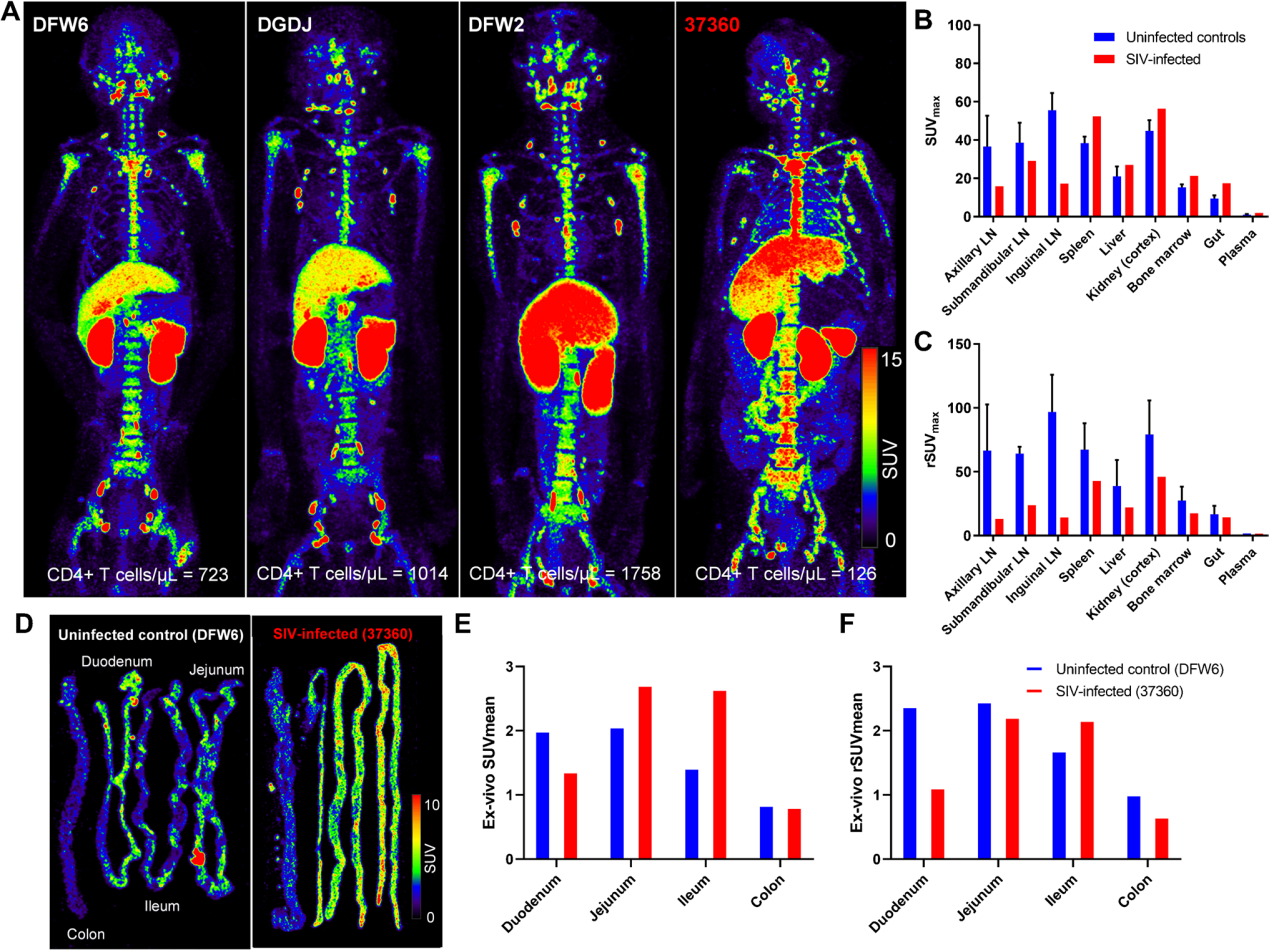
Maximum intensity projection in vivo PET images of rhesus macaques and ex vivo PET images of small and large intestines following administration of 1000 μg mass of [^89^Zr]CD4R1-F(ab′)_2_. **A** Maximum intensity projection in vivo PET images of rhesus macaques showing the comparison of radioligand uptake between uninfected controls (DFW6, DGDJ, and DFW2) and SIV-infected animal (37360) scanned at 40 h post-injection. Tissue uptakes were converted to RAINBOW color map as shown in the color bar, where red color indicates the high standardized uptake value (SUV). Comparison of maximum SUV (SUVmax) in tissues and SUV in the plasma (**B**), and blood pool adjusted SUVmax (rSUVmax) (**C**) in tissues and plasma between uninfected controls (blue, *n* = 3) and SIV-infected animal (red, *n* = 1) at 40 h post-injection. Plasma SUV was calculated from the gamma counter. Plots are mean values and error bars are standard deviation. Maximum intensity projection ex vivo PET images of small and large intestines showing radioligand uptake comparison in the gut sections between one uninfected control (DFW6) and one SIV-infected animal (37360) euthanized at 42–45 h post-injection (**D**) and their SUV (**E**, **F**). The average gut SUV_mean_ was ~ 20-fold lower than the spleen SUV_mean_ in the uninfected control

Byrareddy et al. [12] and Santangelo et al. [13] used 1000 μg mass of the same F(ab΄)_2_ fragment of CD4R1 mAb (tenfold higher mAb mass than what we administered in our earlier studies) radiolabeled with the ^64^Cu positron emitter, performed static PET scans at 24–48 h p.r.i, and reported high radiotracer uptake throughout the GI tract of healthy uninfected animals and differences in gut uptake with varying PB CD4 + T-cell counts. Given that difference in the mass of the administered probe might be one explanation for the observed differences in the gut, we administered ~ 1000 μg of [^89^Zr]CD4R1-F(ab΄)_2_ to four macaques. The biodistribution at 40 h p.r.i in the uninfected controls (CD4^+^ T-cell counts: 1165 ± 534/μL) and SIV-infected animal (CD4^+^ T-cell counts: 126/μL) revealed low uptake in the gut (Fig. 2), similar to the high specific condition of radiotracer administration and consistent with our earlier observations [10, 11] indicating that differences in gut uptake between our studies and [^64^Cu]Cu-CD4R1-F(ab΄)_2_ studies [12, 13] were not due to the mass of the probe. Of note, splenic uptake (Fig. 2A, B, and S4B) in the infected monkey (*n* = 1) was higher than in uninfected controls. The reason for this is unclear but underscores the semi-quantitative nature of SUV measurements, which are obtained from a single static scan, a net signal at the time of image acquisition that disregards the differential blood flow and clearance of radioligand between animals. But when the tissue uptakes were adjusted for blood SUV, the uptake ratio of spleen-to-blood was, as expected, higher in uninfected than in SIV-infected (Fig. 2C and Table S2).

**Fig. 3:**
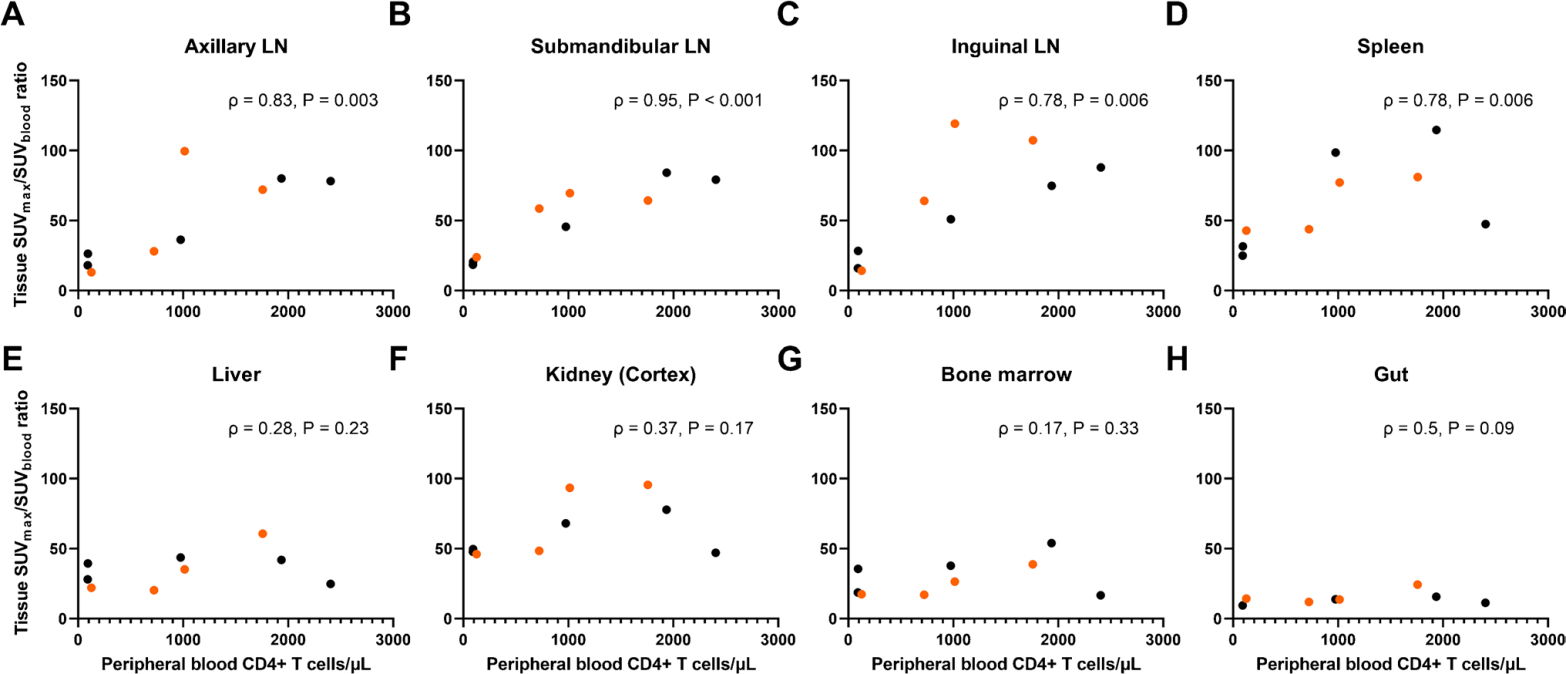
Relationship between peripheral blood CD4 + T-cell counts and maximum tissue-to-blood SUV ratio from PET images obtained from all 9 animals imaged with [^89^Zr]CD4R1-F(ab′)_2_ at 40–48 h post-administration (black dots 100 μg mass and orange dots 1000 μg mass). The relationship was assessed with Spearman rank correlation

We then assessed the relationship between PB CD4 + T-cell counts and radiotracer uptake from the 9 animals imaged with [^89^Zr]CD4R1-F(ab′)_2_ at 40–48 h post-administration regardless of the mAb mass (Fig. 3). We observed strong significant correlations with the spleen and clusters of lymph nodes (*ρ* ≥ 0.78, *P* < 0.01), but not with the other tissues, with strengths of correlations similar to our previous SPECT studies with intact OKT4A, or the F(ab′)_2_ fragments of OKT4A and CD4R1 mAbs [9, 11].

### Ex vivo imaging

The [^89^Zr]CD4R1-F(ab΄)_2_ uptake in the gut and lymphoid organs was further investigated by euthanizing two sets of animals (Fig. S1) following radiotracer injection. One uninfected and one SIV-infected animal, who received 1000 μg of [^89^Zr]CD4R1-F(ab΄)_2_, were euthanized at 42–45 h p.r.i. After flushing the feces out, large sections of the duodenum, jejunum, ileum, and colon, representing ~ 50% (based on weight) of the entire small and large intestines, were PET imaged (Fig. 2D). These ex vivo images did not reveal evidence of higher radiotracer uptake in the gut of the uninfected compared to the SIV-infected animal (Fig. 2E–F) and the SUVs were consistent with measurements of small random aliquots from each gut section counted on a gamma counter (SUV range 0.7–2.2). The average gut SUV_mean_ was ~ 20-fold lower than the spleen SUV_mean_ in the uninfected control. Also, within each animal, the CPM/g of abdominal LNs (mesenteric and retroperitoneal) was similar to the CPM/g measured in peripheral LNs (axillary, submandibular, and inguinal) (data not shown). Similar conclusions were reached by counting random aliquots of each gut section (SUV range 0.4–0.8) and lymphoid tissues on a gamma counter in another uninfected and SIV-infected animal pair that received 100 μg of [^89^Zr]CD4R1-F(ab΄)_2_ (Fig. S1) and euthanized at 145–147 h p.r.i. (data not shown).

### PET imaging with [^89^Zr]ibalizumab

**Fig. 4:**
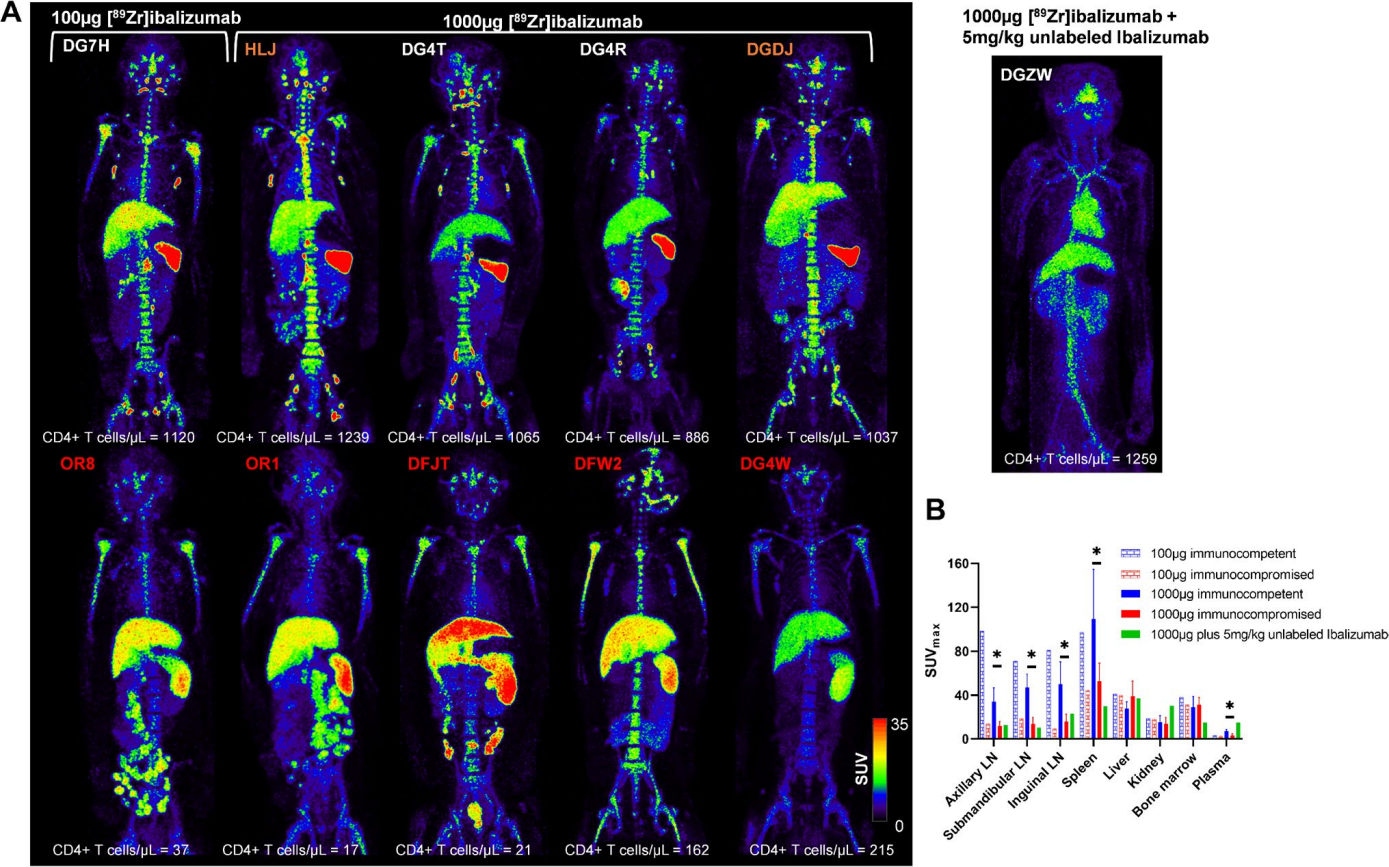
[^89^Zr]ibalizumab biodistribution at 40–48 h post-injection. **A** Maximum intensity projection in vivo PET images of rhesus macaques showing the comparison of radioligand uptake between immunocompetent (DG7H, HLJ, DG4T, DG4R, and DGDJ) and immunocompromised animals (OR8, OR1, DFJT, DFW2, and DG4W) following administration of 100 μg (DG7H and OR8) or 1000 μg mass (HLJ, DG4T, DG4R, DGDJ, OR1, DFJT, DFW2, and DG4W) of [^89^Zr]ibalizumab and scanned at 40–48 h post-injection. Radiotracer specific binding to CD4 was demonstrated by competition in one immunocompetent uninfected control (DGZW) by co-injecting 1000 μg of [^89^Zr]ibalizumab with 5 mg/kg of unlabeled Ibalizumab. Tissue uptakes were converted to RAINBOW color map as shown in the color bar, where red color indicates the high standardized uptake value (SUV). **B** Comparison of maximum SUV (SUVmax) in tissues and SUV in the plasma between immunocompetent (100 μg: blue, pattern, *n* = 1; 1000 μg: blue, solid, *n* = 4), immunocompromised (100 μg: red, pattern, *n* = 1; 1000 μg: red, solid, *n* = 4), and CD4-block with excess unlabeled ibalizumab animals (green, *n* = 1) at 40–48 h post-injection. Plots are mean values and error bars are standard deviation. Statistically significant differences in radiotracer uptakes (indicated by * for *P*-value < 0.05) were observed in lymph nodes, spleen, and plasma between the immunocompetent and immunocompromised animals that received 1000 μg of [^89^Zr]ibalizumab. Note that competition with unlabeled ibalizumab results in blocking of uptake from the spleen and lymph nodes leading to higher circulation of tracer in the blood (heart pool)

We further corroborated the in vivo observations of CD4R1-F(ab΄)_2_ mAb by imaging with ibalizumab, a recombinant humanized anti-CD4 IgG4 mAb in 11 animals (Fig. S1). Similar to [^89^Zr]CD4R1-F(ab΄)_2_, we observed high specific binding of [^89^Zr]ibalizumab to MT4 cells (Fig. S2D). PET/CT imaging of [^89^Zr]ibalizumab was performed at 40–48 h and 134–144 h p.r.i using 100 μg (high specific activity) or 1000 μg (low specific activity) of ibalizumab in five pairs of animals (Figure S1), each pair consisting of an immunocompetent and an immunocompromised macaque. Four of the five immunocompromised SIV-infected macaques (OR8, OR1, DFJT, and DG4W) received C207, an anti-CD3 immunotoxin, on two consecutive days (total 4 doses; each dose 50 μg/kg) 5–7 days before radiotracer injection to further deplete CD4 + T-cells [26]. The PET images of [^89^Zr]ibalizumab showed similar biodistributions to [^89^Zr]CD4R1-F(ab΄)_2_ in the spleen and clusters of lymph nodes and a statistically significant difference (*P* < 0.05, *n* = 4 vs. 4, 1000-μg dose group) in these organs uptake between immunocompetent and immunocompromised macaques (Fig. 4, S6 and S7). Some discrete focal intraluminal uptake was noted in the gut at 40–48 h p.r.i but completely cleared at 134–144 h p.r.i, indicating the non-specific nature of the radiotracer retention in the gut foci. Minimal kidney uptake was observed. Moreover, ibalizumab radiolabeled at low specific activity was near 100% stable in PBS, human, and monkey plasmas at 37 °C for up to 40 h (Fig. S2E), as well as in vivo at 40 h p.r.i as shown in Fig. S2F for 4 animals belonging to the 1000-μg dose group.

Additionally, to demonstrate radiotracer specificity in vivo, we next performed a CD4-blocking study in one immunocompetent uninfected monkey (DGZW) by co-injecting 1000 μg of [^89^Zr]ibalizumab with 5 mg/kg of unlabeled unconjugated Ibalizumab. Both visual and semi-quantitative analysis of the PET revealed a dramatic reduction of radiotracer uptake in the spleen and the clusters of lymph nodes compared to [^89^Zr]ibalizumab alone (Fig. 4 and S6). Thus, the ibalizumab-radiolabeled probe offers a candidate tracer for potential translation of the CD4-pool imaging system to human studies.

### Binding potential

**Fig. 5:**
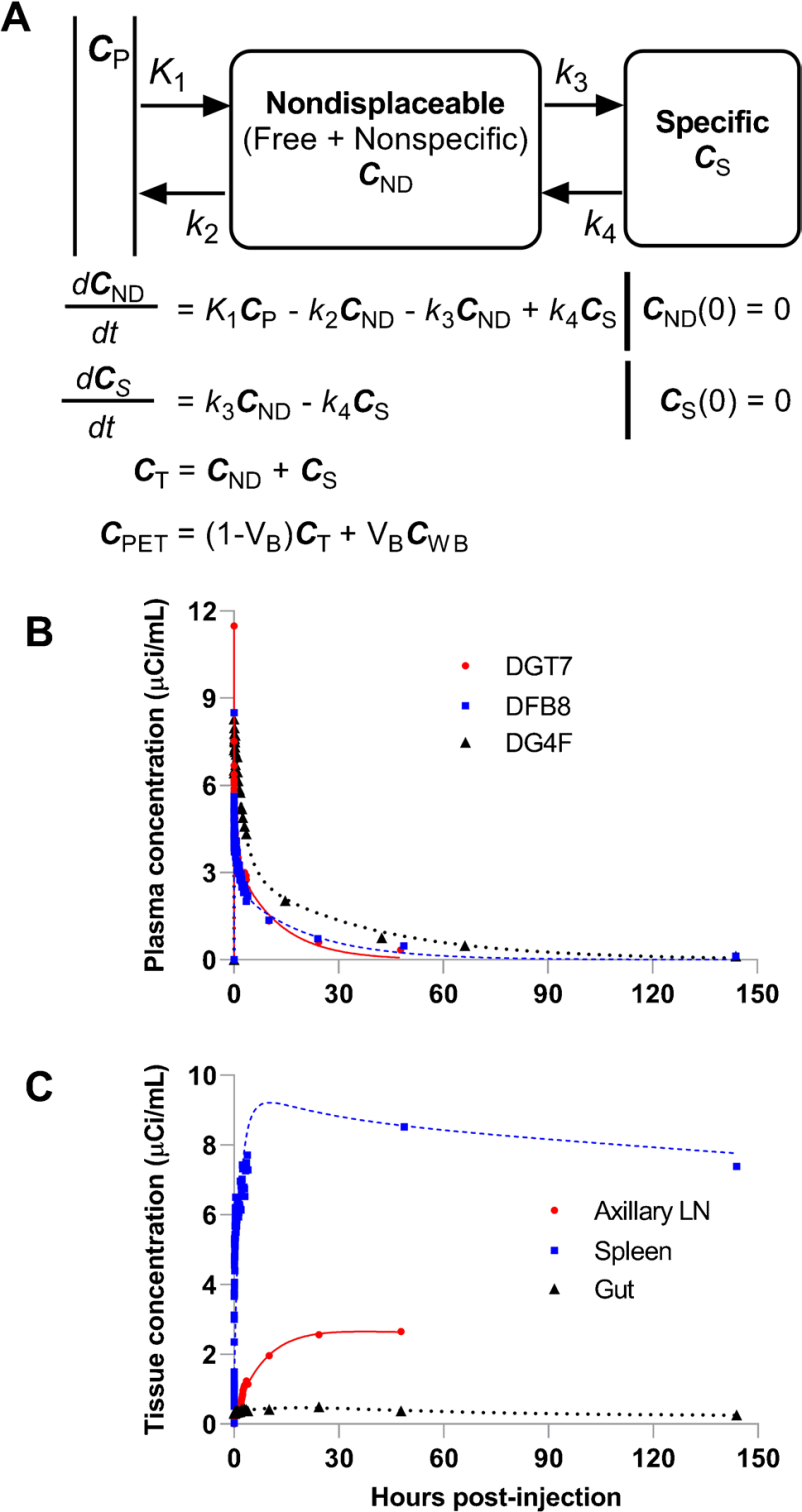
**A** Two-tissue compartment ligand-receptor model. Nondisplaceable (C_ND_) tissue compartment encloses the free ligand in tissue plus ligand bound to non-specific sites, and the specific (C_S_) tissue compartment represents ligand bound specifically to receptors. Metabolite-corrected (free parent) arterial plasma (C_P_) is the input to the tissue. Rate constants K_1_ and k_2_ describe the transfer of ligand from plasma to tissue and vice versa, and k_3_ and k_4_ describe the transfer of ligand between nondisplaceable and specifically bound ligand in tissue. V_B_ is the regional blood volume per unit tissue, C_WB_ is the whole-blood ligand concentration, and C_PET_ is the tissue concentration measured from PET image. **B** Metabolite-corrected radioligand concentration in plasma following an intravenous bolus injection of [^89^Zr]CD4R1-F(ab′)_2_ in three uninfected controls, DGT7 (red circle and solid line), DFB8 (blue square and dashed line), and DG4F (black triangle and dotted line) interpolated with best fitting a 3-exponential decay model. **C** Time-activity curves of concentration of [^89^Zr]CD4R1-F(ab′)_2_ in axillary lymph node (red circle and solid line), spleen (blue square and dashed line), and the gut (black triangle and dotted line) following an intravenous bolus radioligand injection best fitted to the two-tissue compartment model

While simplistic approaches of qualitative (visual) and semi-quantitative measures (SUV or tissue-to-blood SUV ratios) from a single static scan are valuable, dynamic scanning with kinetic analyses provides a superior and fully quantitative measure of receptor concentration in a given organ [18]. By measuring the time course of input of free ligand into the tissue, a dynamic scan to tease out from the measured PET signal the contributions of whole blood, the free ligand in tissue, the non-specifically and specifically bound ligand in tissue, and using a two-tissue compartment model [27] (Fig. 5A), we next estimated the in vivo binding potential for CD4 receptors in the LN, spleen, and the gut of uninfected controls in which the ligand was administered in tracer doses (~ 100 μg; 2–3 log lower than pharmacologically therapeutic dose). The in vitro binding potential (BP) is the product of receptor density (B_max_; units: nmol/L (nM)) and receptor affinity (K_a_ = 1/K_d_, where K_d_ (nmol/L) is the radioligand equilibrium disassociation constant, units: nM^−1^) [30]. Assuming all receptors across various tissues have the same affinity for the radioligand, the binding potential measure thus reflects tissue receptor density.

An estimate of LN or spleen in vitro BP can be derived from some of our earlier work in which we have isolated CD4^+^ and CD4^−^ cells with magnetic beads from LNs and spleen of macaques after administration of a radiolabeled anti-CD4 probe. From those experiments, we estimated similar concentrations of CD4^+^ T-cells in the spleen and LN in healthy adult rhesus macaques, an average of 1.25 × 10^9^ CD4^+^ T-cells per gram [9]. Using a mean number of 75,000 receptors per CD4^+^ T-cell [31] and a specific volume of 0.96 mL/g of tissue [28], we calculated ~ 162 nM as the theoretical concentration of the CD4 receptors in the spleen or LN. As a one log range of K_d_ (0.3–2.5 nM) was reported from OKT4A binding studies to the CD4 receptor [9, 21], the in vitro BP is expected to fall in the range of 65–540. BP_F_ is an in vivo measurement that reflects BP (B_max_/K_d_) and is defined as B_avail_/K_d_, where B_avail_ (nmol receptor per 1000 cm^3^ tissue) is the receptor density (molarity) available for radioligand binding, which for radioligand administered at tracer doses is close to B_max_. The BP_F_ is also equivalent to the ratio at equilibrium of the concentration of specifically bound radioligand in tissue (C_S_) to the free radioligand concentration in plasma (C_P_) and can be expressed in terms of kinetic rate constants as (K_1_*k_3_)/(k_2_*k_4_) by solving the model equations at equilibrium.

To track the rapid initial changes in the concentration of the probe in the blood, plasma, and tissue following a bolus injection, we performed a dynamic scan for the first 4 h p.r.i. The regional blood volume (V_B_) values of 0.05 and 0.34 were fixed for axillary LN and spleen, respectively [28]. As the transfer rate of the ligand from specific binding sites (k_4_) could be assumed equal among the tissues, coupling k_4_ among the studies, 11 parameters (K_1_, k_2_, and k_3_, for each of 3 tissues (spleen, axillary LN, and the gut), V_B_ for the gut, and a common k_4_) were estimated at once by simultaneously best fitting the “mean” tissue concentration of the spleen, axillary LN, and the gut obtained from each frame of the dynamic scan and from the static scans obtained at later time points to the model. To avoid local minima, we performed 1000 random fits with randomized sets of initial parameters and obtained the best-fit parameters resulting in a global minimal chi-square. We estimated a common rate constant of 1.76 × 10^−5^ min^−1^ (95% confidence interval: 1.48–2.04 × 10^−5^ min^−1^) for k_4_, a BP_F_ of ~ 185 mL·cm^−3^ (95% confidence interval: 165–204 mL·cm^−3^) in LN tissue, and ~ 263 mL·cm^−3^ (95% confidence interval: 253–274 mL·cm^−3^) in the spleen (Fig. 5 and Table 1).

**Table 1:** Parameter estimates and percent standard error (SE) of K_1_, k_2_, k_3_, and BP_F_ using a two-tissue compartment model

Tissue	K_1_ (mL·cm^−3^·min^−1^)		k_2_ (min^−1^)		k_3_ (min^−1^)		BP_F_ (mL·cm^−3^)	
	Value	%SE	Value	%SE	Value	%SE	Value	95% Confidence interval
Lymph node	0.00154	2.5	4.80E-04	20.7	0.00101	17.5	184.9	165–204
Spleen	0.0302	4.8	0.005	8.8	7.69E-04	5.5	263.4	253–274
Gut	2.06E-04	6.2	9.91E-04	11.5	2.18E-04	10.7	2.6	2.3–2.9

For large tissues such as the spleen and liver, the in vivo SUV_mean_ from PET image and ex vivo SUV (calculated using CPM/g from gamma counter) were similar. However, small tissues such as lymph nodes quantitatively suffered from partial volume effect in their value of in vivo SUV_mean_ and were underestimated as compared to ex vivo (gamma counter generated) SUV by as much as 40%. We made no quantitative correction of this underestimation in the LN tissue concentration used in best fitting, and as such, the BP_F_ estimate of LN was assumed to be an underestimation. We estimated a V_B_ of 0.071 (95% confidence interval: 0.065–0.078) and a BP_F_ of 2.6 mL·cm^−3^ (95% confidence interval 2.3–2.9) for the gut, an estimate that is at least tenfold lower than the spleen’s BP_F_ estimate. Given that the weight of the spleen (150 g) [28] is approximately tenfold lower than the weight of the total gut in the adult human male (1200–1500 g) [28, 32], it can be conservatively concluded that the contribution of the intestines to the total CD4 T-cell pool in the body is lower than the contribution of the spleen alone.

## Discussion

In our previous work using SPECT technology [9–11], we noninvasively imaged the CD4 pool in vivo in nonhuman primates with a tracer mass (~ 100 μg) of a humanized anti-CD4 intact mAb (OKT4A), the F(ab′)_2_ fragment of OKT4A, or the F(ab*′*)_2_ fragment of a rhesus recombinant anti-CD4 mAb (CD4R1), labeled with the single-photon gamma emitter (^111^In or ^99m^Tc). Imaging up to 96 h p.r.i with the intact and up to 21 h p.r.i with the F(ab*′*)_2_ fragment anti-CD4 probes, we showed that, in quasi steady state, the PB CD4 + T-cell count is significantly correlated with the size of the spleen, axillary, inguinal, and submandibular LNs CD4 pool [9, 11]. With ex vivo studies of CD4 + sorted cells coupled with the observed differences in radiotracer uptake between healthy and CD4-depleted animals, we also showed that most of the uptake in these anatomic compartments was the result of specific binding of the probe to CD4 receptors. Despite > 1 log_10_ difference in PB CD4 + T-cell counts among the imaged monkeys, both in vivo SPECT images and ex vivo radioactivity data obtained at 21 h p.r.i revealed no differences in the radiotracer uptake of the gastrointestinal tract between uninfected and SIV-infected CD4-depleted animals, which in all animals was exceptionally low compared to the uptake in the spleen and LNs.

One hypothetical reason for the meager gut uptake was that the biodistribution into the gut would need a longer time. The relatively short half-life of ^99m^Tc (6.01 h) precluded our previous SPECT scans to be obtained later than ~ 21 h p.r.i. In the current study, the relatively long half-life of ^89^Zr allowed to extend the biodistribution time and obtain high-quality images even at 144 h (6 days) p.r.i. Using a PET camera, we observed specific uptake of the anti-CD4 probe [^89^Zr]CD4R1-F(ab′)_2_ in the spleen and clusters of LNs, but scant uptake in the gut at both 100 μg and 1000 μg mAb dose groups, which appears non-specific. Based on our imaged animals and sample size, both visual and semi-quantitative uptake measures (SUV and tissue-to-blood SUV ratio) for each tissue in both the 100-μg or 1000-μg dose groups were comparable and there appears to be no advantage in either increasing the mass of the F(ab΄)_2_ probe to 1000 μg or extending the biodistribution time to 144 h (6 days). Furthermore, both ^89^Zr-radiolabeled CD4R1-F(ab΄)_2_ and ibalizumab showed similar high uptakes in LNs and spleen, and low uptakes in the gut.

While measurement strategies such as cell extraction procedures or ex vivo tissue staining can provide quantitative and/or qualitative measures of receptor densities on a micro-level, which can then be extrapolated using specific assumptions to the overall organ, in vivo imaging technologies have a tremendous advantage over these techniques by overcoming the intrinsic limitations of approaches that suffer from the tissue cell extraction yield, the efficacies of tissue staining or antigen retrievals from tissue slices which vary based on storage and processing conditions, and sampling bias due to tissue heterogeneity. The two-compartment kinetic modeling of in vivo PET imaging data described in our study can provide, in addition to the visual and simplified semi-quantitative measurements, an unparalleled fully quantitative measure of organ-specific receptor density on a macro-level; however, within the underlined assumptions, which in our CD4-pool PET imaging study relate to (1) probe affinity to the CD4 receptor is not anatomic compartment-specific, that (2) most of the specific component of the tissue time-activity curve is the result of binding to CD4 + T-cells [9, 33], (3) the levels of CD4 expression per CD4 + T-cell are similar among tissues as suggested from mean fluorescence intensity analyses of CD4 + T-cells extracted from organs [34, 35], that (4) the probe is injected at tracer doses [27, 33], and finally, (5) the blood flow and the diffusion of the probe into the tissue must be sufficient to generate an observable binding potential. Under the aforementioned assumptions, the quantitative measure of receptor densities estimated in this in vivo study, when translated from macaque levels to human proportions, unraveled the relative size of the gut CD4 pool to be lower than that of the splenic CD4 pool and thus challenging the conventional wisdom in this area.

Additionally, our findings are also consistent with CD4 and CD8 immuno-PET biodistribution studies in rodent models, which revealed through blocking (with excess unlabeled mAb) and depletion experiments, a specific uptake of the radiotracer (^89^Zr-radiolabeled anti-CD4 or anti-CD8 fragments) only in the LNs and spleen [36–38]. Furthermore, human studies of ^89^Zr anti-CD8 minibody showed high uptake in the spleen with little uptake in the bowel that was consistent with intraluminal activity [39]. Previous studies imaging cutaneous T-cell lymphoma with [^111^In]In-T101, an anti-CD5 labeled antibody, showed prominent uptake in the spleen and nodes but no specific localization in the bowel [40]. Of note, the PET imaging of programmed death-1 (PD-1) + cells, which represent approx. 30–50% of PBMC and LN cells in healthy nonhuman primates [41] also showed evidence of specific uptake only in the spleen and the LNs [42, 43].

## Conclusion

This study has provided the first to our knowledge estimate of the CD4 binding potential in the spleen and LNs from in vivo PET dynamic imaging and demonstrated that the gut is not the predominant reservoir of CD4 + T-cells in nonhuman primates and contributes no more, and likely considerably less than that of the spleen. These data further inform on the relative contributions of predominant lymphoid tissues and the gut to the overall CD4 + T-cell pool and should facilitate studies seeking to examine HIV pathogenesis and treatment strategies directed at viral eradication.

## Supplementary Information

Below is the link to the electronic supplementary material.

Supplementary file1 (DOCX 2.59 MB)

Supplementary file2 (MP4 4609 KB)

Supplementary file3 (MP4 3904 KB)

## Data Availability

Data will be made available upon reasonable request.
