# Supplementary material for: Quantitative PET imaging of the CD4 pool in nonhuman primates
Source: Eur J Nucl Med Mol Imaging. 2022 Aug 27;50(1):14–26. doi: 10.1007/s00259-022-05940-4 (PMC9668939; doi:10.1007/s00259-022-05940-4)
Supplement: Supplementary file 1 — Supplementary file1 (DOCX 2.59 MB) [file 259_2022_5940_MOESM1_ESM.docx]

**Quantitative PET imaging of the CD4 pool in nonhuman primates**

**Insook Kim^1^**†**, Sharat Srinivasula^2^**†**, Paula DeGrange^3^, Brad Long^3^, Hyukjin Jang^2^, Jorge A. Carrasquillo^4,5^, H. Clifford Lane^6^, Michele Di Mascio^7^***

^1^AIDS Imaging Research Section, Applied/Developmental Research Directorate, Frederick National Laboratory for Cancer Research; Frederick, MD 21702, USA.

^2^ AIDS Imaging Research Section, Clinical Monitoring Research Program Directorate, Frederick National Laboratory for Cancer Research; Frederick, MD 21702, USA.

^3^ AIDS Imaging Research Section, Charles River Laboratories, Integrated Research Facility, NIAID, NIH; Frederick, MD 21702, USA.

^4^ Molecular Imaging and Therapy Service, Radiology Department, Memorial Sloan Kettering Cancer Center, New York, NY 10065, USA.

^5^ Molecular Imaging Branch, Center for Cancer Research, NCI, NIH; Bethesda, MD 20892, USA.

^6^ Laboratory of Immunoregulation, Division of Intramural Research, NIAID, NIH; Bethesda, MD 20892, USA.

^7^ AIDS Imaging Research Section, Division of Clinical Research, NIAID, NIH; Bethesda, MD 20892, USA.

*Corresponding author. **Michele Di Mascio**

AIDS Imaging Research Section, Division of Clinical Research, NIAID, NIH

5601 Fishers Ln, Rm 4D20, Rockville, MD 20852, USA

Tel: 1 301-793-2785; e-mail: [mdimascio@niaid.nih.gov](mailto:mdimascio@niaid.nih.gov)

†These authors contributed equally to this work.

1. **Supplemental Materials and Methods**

*Preparation of* *F(ab*′*)_2_*. We prepared F(ab′)_2_-CD4R1 by pepsin digestion according to the

manufacturer’s instruction (Pierce F(ab’)_2_ Preparation Kit, Thermo Fisher Scientific). Briefly, 20 mg of CD4R1 (5.1 mg/ml) was digested by pepsin immobilized on agarose gel (Thermo Fisher Scientific) in 0.1M sodium acetate buffer (pH 4.4). The digestion was performed at 37°C for 6h using an optimal antibody/pepsin weight ratio between 3.5-5:1. After digestion, the reaction mixture was purified using Protein-A Sepharose affinity chromatography (Thermo Fisher Scientific), followed by dialysis in PBS using a dialysis membrane cassette with 20 kDa molecular weight cut-off at 4°C for 22h. The purified protein was concentrated using a Centriprep YM-50 membrane (Amicon) to a concentration of 13 mg/ml. Protein purity was assessed by SDS-PAGE (Invitrogen) and size-exclusion HPLC (Gilson, Middleton, WI) equipped with a size-exclusion TSK gel G3000SWxL column (7.8 × 300 mm, 5 μm, TOSOH Bioscience; 0.067M sodium phosphate/0.15M sodium chloride with 0.1M KCl, pH 6.8; 1.0 ml/min) and a UV monitor.

*Conjugation of p-SCN-Df to F(ab´)_2_-CD4R1 and intact ibalizumab.* The bifunctional chelating agent p-isothiocyanatobenzyldesferrioxamine (Df-Bz-NCS) was conjugated with lysine residues of F(ab′)_2_-CD4R1 or Ibalizumab to produce Df-F(ab′)_2_-CD4R1 or Df-Ibalizumab, respectively following a method of Vosjan et al.[1]. Briefly, fragmented or intact antibody (mAb) was reacted with p-SCN-Df at a molar ratio of 1:3 in 0.1M sodium bicarbonate, at pH 9.5 at 37°C. The Df-conjugated mAbs were purified with a Zeba Spin Desalting column (7K MWCO, Pierce Biotechnology, Rockford, Illinois) in 0.25M sodium acetate buffer. The column was pretreated with 0.25M sodium acetate buffer.

*Immunoreactivity binding assays.* The immunoreactivity binding of [^89^Zr]CD4R1-F(ab*´*)_2_ and [^89^Zr]ibalizumab was tested in MT4 cells using a modification of the saturation cell-binding assay under antigen excess conditions using the method of Lindmo et al. [2], as previously described [3, 4].

*Plasma assay and radio-HPLC.* Before radioligand administration, any pre-existing immune response against CD4R1 or Ibalizumab mAb was tested using a plasma binding assay coupled with radio-HPLC analysis, as previously described [5]. Briefly, rhesus plasma was incubated with [^89^Zr]CD4R1-F(ab΄)_2_ or [^89^Zr]ibalizumab (incubation concentration 1.5nM) for 30 min at 37°C in a humidified 5% CO_2_ incubator. After the incubation, a 50µl aliquot of the incubation mixture was run through size-exclusion HPLC. A 20µl aliquot was added to 2 million viable MT4 cells in 180µL (incubation concentration 0.15nM) and incubated at 4°C for 90 min. The total incubated radioactivity counts per minute (CPM) were measured in a gamma counter (PerkinElmer 1480 Wizard or 2480 Wizard2), and the cell mixtures were microcentrifuged at 12,000 rpm for 5 min (Eppendorf 5415C). After the supernatant was aspirated and discarded, the CPM in the cell pellet was measured, and the percent of total incubated radioactivity bound to MT4 cells was determined.

*PET/CT Imaging.* Imaging was performed using a novel PET/CT camera designed for nonhuman primates (MultiScan LFER 150 PET/CT, Mediso, Hungary). Animals were initially anesthetized with a restraint dose of ketamine (10 mg/kg). After shaving the skin and prepping the insertion sites, a 22-gauge catheter was inserted in the saphenous vein of the leg for bolus injection of radiotracer or continuous administration of anesthetics (propofol 0.2 mg/kg/min infusion) during the imaging procedures. The arms and legs of the macaque were restrained and positioned in supine orientation for imaging. Anesthetized primates were monitored with a pulse oximeter and thermometer, and the body temperature during imaging was maintained with Bair Hugger patient warming system.

For dynamic scans, immediately following a whole-body CT scan, animals underwent a 4-hour PET scan commenced with bolus radioligand injection with either the bed fixed at one field-of-view (FOV) (for animal DGT7: containing axillary lymph nodes (LNs) and heart in the FOV; for animal DFB8: containing heart and spleen in the FOV) or the bed moving to cover multiple-FOVs (for animal DG4F: encompassing axillary LN, heart, spleen, and the gut). For static scans, immediately following a whole-body CT scan, animals underwent a whole-body PET scan from the top of the head to mid-thighs at one or multiple time points post-injection as shown in the imaging schema (Fig S1). Static scans were acquired at 10 min per FOV, and depending on the animal’s length, 5 or 6-bed positions were scanned with a 40% overlap between the FOVs. Raw CT data was reconstructed with scatter correction. Raw PET data was reconstructed using Tera-Tomo 3D reconstruction at 1mm voxel size for 8 iterations and 9 subsets. Reconstructed images were corrected for attenuation (using CT), radioactive decay, uniformity, random coincidences, scattering of radiation, and the decay reference was set to radioligand administration time. A Gaussian post-processing filter with kernel size = 3 and sigma = 0.8 was applied to smooth the reconstructed PET image. Final CT and PET images were saved in DICOM format. In addition to a whole-body CT scan on PET/CT, animals underwent additional high-resolution CT scans on Symbia T2 SPECT/CT camera (Siemens) on the same day of PET imaging.

*Data Analysis.* PET image analysis was performed using MIM software (version 6.9.2, Cleveland, USA). [^89^Zr]CD4R1-F(ab΄)_2_ and [^89^Zr]ibalizumab uptakes of selected volumes of interest (VOI) were identified on CT scan (Fig S8). VOI were manually drawn on the PET image for axillary, inguinal, and submandibular LNs, spleen, liver, right-kidney, bone marrow, and the gut. The amount of radioligand uptake was quantified either as decay-corrected tissue concentration (µCi/mL) or standardized uptake value (SUV) using the formula: SUV = (*c/d)*w*, where *c* is decay-corrected tissue concentration (µCi/mL), *d* is the injected dose (µCi), and *w*, the body weight (g). The SUVmax value as a measure of maximum radioligand concentration and SUVmean as a measure of average radioligand uptake in the 3-dimensional VOI were calculated from PET images. To account for differences among the animals in the clearance of the radiotracer, we also calculated the SUVmax in tissue relative to blood SUVmean (rSUVmax). Bone marrow VOI was placed on the proximal segment of the humerus. Care was taken to avoid any mesenteric LNs in the gut VOIs. At each imaging time point, whole blood and plasma SUV were calculated by drawing blood and counting aliquots in the gamma counter. Images and the quantitative measures were not corrected for partial volume effect.

1. **Supplemental Results**

**Consideration of alternate kinetic models**

In addition to the 2-tissue compartment (2TC) model of 5 parameters used to best fit the PET image data, we have explored two other models. The first is a 3-tissue compartment (3TC) model of 7 parameters introduced by Fujita et al [6] which accounts for a labeled metabolite of the radiotracer entering the tissue and undergoing non-specific binding (Figure S10A). Though we did not observe any metabolite formation of our radiotracer from radio-HPLC of the *in-vivo* plasma, we assumed an input function in which metabolites start to form *in-vivo* soon after the radiotracer injection with ~50% of the plasma input at 24-48h after radiotracer administration explained by metabolites, and the metabolite fraction in the plasma growing to ~90% at 144h (6 days). By best fitting the tissue time-activity curves to this 3TC model of 7 parameters, we estimated a spleen BP_F_ of 314 (95% confidence interval 305-323) and gut BP_F_ of 3.67 (95% confidence interval 3.1-4.24).

The second model explored is a 3-tissue compartmental model of 7 parameters that accounts for radioligand internalization (Figure S10B). Here, after the specific binding of the radioligand to the receptor, the ligand-receptor complex is internalized and contributes to the PET signal. At ~144h post-radiotracer administration, we assumed that ~30% of the specific binding is explained by the internalized signal. Using this model, we estimated a spleen BP_F_ of 237 and gut BP_F_ of 2.2. In other words, the minimalistic 2TC model in which the internalization component is absent, overestimates, as expected, the binding potential of ~10-20%, but does so for both tissues, hence without affecting the conclusion.

With both models explored, we estimated spleen BP_F_ to be 10-100 fold higher than the gut BP_F_.

**References**

1. Vosjan MJ, Perk LR, Visser GW, Budde M, Jurek P, Kiefer GE, et al. Conjugation and radiolabeling of monoclonal antibodies with zirconium-89 for PET imaging using the bifunctional chelate p-isothiocyanatobenzyl-desferrioxamine. Nat Protoc. 2010;5:739-43. doi:10.1038/nprot.2010.13.

2. Lindmo T, Bunn PA, Jr. Determination of the true immunoreactive fraction of monoclonal antibodies after radiolabeling. Methods in enzymology. 1986;121:678-91.

3. Di Mascio M, Paik CH, Carrasquillo JA, Maeng JS, Jang BS, Shin IS, et al. Noninvasive in vivo imaging of CD4 cells in simian-human immunodeficiency virus (SHIV)-infected nonhuman primates. Blood. 2009;114:328-37. doi:10.1182/blood-2008-12-192203.

4. Di Mascio M, Srinivasula S, Kim I, Duralde G, St Claire A, DeGrange P, et al. Total body CD4+ T cell dynamics in treated and untreated SIV infection revealed by in vivo imaging. JCI Insight. 2018;3:e97880. doi:10.1172/jci.insight.97880.

5. Srinivasula S, Gabriel E, Kim I, DeGrange P, St Claire A, Mallow C, et al. CD4+ levels control the odds of induction of humoral immune responses to tracer doses of therapeutic antibodies. PLoS One. 2017;12:e0187912. doi:10.1371/journal.pone.0187912.

6. Fujita M, Seibyl JP, Verhoeff NP, Ichise M, Baldwin RM, Zoghbi SS, et al. Kinetic and equilibrium analyses of [(123)I]epidepride binding to striatal and extrastriatal dopamine D(2) receptors. Synapse. 1999;34:290-304. doi:10.1002/(SICI)1098-2396(19991215)34:4<290::AID-SYN5>3.0.CO;2-B.

**Table S1** Characteristics of animals used in the study

| **Antibody** | **ID** | **Infection** | **Gender** | **Age (Y)** | **CD4+ T cells/µL** | **Plasma viral load (copies/mL)** | **mAb mass**  **(µg)** | **Inj. Activity**  **(MBq)** | **Body weight**  **(kg)** | **mAb mass/**  **body weight (µg/kg)** |
| --- | --- | --- | --- | --- | --- | --- | --- | --- | --- | --- |
| **100µg mAb mass [^89^Zr]CD4R1-F(ab′)2** | DGZW | Uninfected | Male | 3.5 | 2404 | NA | 119 | 57.7 | 6 | 19.8 |
|  | DGT7 | Uninfected | Male | 3.1 | 1935 | NA | 104 | 62.6 | 5.35 | 19.4 |
|  | DFB8 | Uninfected | Male | 5.8 | 975 | NA | 98 | 78.4 | 9.25 | 10.6 |
|  | DG4F | Uninfected | Male | 5.2 | 982 | NA | 126 | 85.8 | 8.25 | 15.3 |
|  | HIH | SIVmac239-nef-stop | Female | 5.9 | 90 | 8.75 x 10^5^ | 122 | 59.8 | 6.4 | 19.1 |
|  | DGKG | SIVmac239-nef-stop | Male | 5.2 | 93 | 3.79 x 10^4^ | 89 | 68.5 | 8.05 | 11 |
|  |  |  |  |  |  |  |  |  |  |  |
| **1000µg mAb mass [^89^Zr]CD4R1-F(ab′)2** | DFW6 | Uninfected | Male | 6.0 | 723 | NA | 900 | 115.9 | 7.95 | 113.2 |
|  | DGDJ | Uninfected | Male | 4.9 | 1014 | NA | 827 | 126.9 | 7.1 | 116.5 |
|  | DFW2 | Uninfected | Male | 6.2 | 1758 | NA | 834 | 126.9 | 6.9 | 120.9 |
|  | 37360 | SIVmac239-nef-stop | Female | 13.7 | 126 | 1.63 x 10^5^ | 900 | 113.6 | 9.25 | 97.3 |
|  |  |  |  |  |  |  |  |  |  |  |
| **100µg mAb mass [^89^Zr]Ibalizumab-intact** | DG7H | Uninfected | Male | 5.3 | 1120 | NA | 121 | 36.3 | 9.2 | 13.2 |
|  | OR8 | SIVmac239-nef-stop | Female | 7.4 | 37 | 2.8 x 10^7^ | 129 | 38.5 | 6.5 | 19.8 |
|  |  |  |  |  |  |  |  |  |  |  |
| **1000µg mAb mass [^89^Zr]Ibalizumab-intact** | HLJ | SIVmac239-nef-stop | Female | 7.7 | 1239 | 9.5 x 10^1^ | 936 | 64.0 | 9.35 | 100.1 |
|  | DG4T | Uninfected | Male | 6.5 | 1065 | NA | 981 | 84.0 | 9.95 | 98.6 |
|  | DG4R | Uninfected | Male | 6.5 | 886 | NA | 990 | 74.2 | 7.6 | 130.3 |
|  | DGDJ | SIVmac251 | Male | 7.2 | 1037 | 8.3 x 10^1^ | 965 | 80.1 | 14.8 | 65.2 |
|  | OR1 | SIVmac239-nef-stop | Female | 7.7 | 17 | 6.25 x 10^5^ | 845 | 61.2 | 5.85 | 144.4 |
|  | DFJT | SIVmac239-nef-stop | Male | 10.2 | 21 | 4.36 x 10^4^ | 973 | 62.6 | 12.35 | 78.8 |
|  | DFW2 | SIVmac239 | Male | 8.5 | 162 | 9.85 x 10^5^ | 959 | 68.7 | 10.35 | 92.7 |
|  | DG4W | SIVmac239 | Male | 6.4 | 215 | 1.93 x 10^6^ | 994 | 77.1 | 10.7 | 92.9 |
|  |  |  |  |  |  |  |  |  |  |  |
| **1000µg mAb mass [^89^Zr]Ibalizumab-intact + 5mg/kg unlabeled Ibalizumab** | DGZW | Uninfected | Male | 6.0 | 1259 | NA | 55727 | 64.9 | 10.95 | 5089 |

**Table S2** Ratio of Tissue SUVmax to whole-blood SUV at 40-48h post-injection of [^89^Zr]CD4R1-F(ab′)_2_

| **Tissue** | 100µg Uninfected | 100µg Infected | 1000µg Uninfected | 1000µg Infected |
| --- | --- | --- | --- | --- |
| **Axillary LN** | 64.9±24.8 | 22.2±5.8 | 66.5±36.1 | 13 |
| **Submandibular LN** | 69.7±21.0 | 19.5±1.5 | 64.1±5.5 | 23.8 |
| **Inguinal LN** | 71.2±18.8 | 22.1±8.8 | 96.9±29.1 | 14.1 |
| **Spleen** | 86.9±35.1 | 28.3±4.7 | 67.4±20.5 | 42.8 |
| **Liver** | 36.8±10.4 | 33.7±8.0 | 38.8±20.5 | 22.1 |
| **Right Kidney** | 64.3±15.7 | 48.7±1.4 | 79.2±26.6 | 46.1 |
| **Bone marrow** | 36.2±18.7 | 27.1±11.9 | 27.4±10.9 | 17.4 |
| **Gut** | 13.6±2.2 | 9.4±0.03 | 16.7±6.7 | 14.3 |
| **Plasma** | 1.9±0.7 | 1.6±0.03 | 1.7±0.02 | 1.6 |

Data are mean±standard deviation.


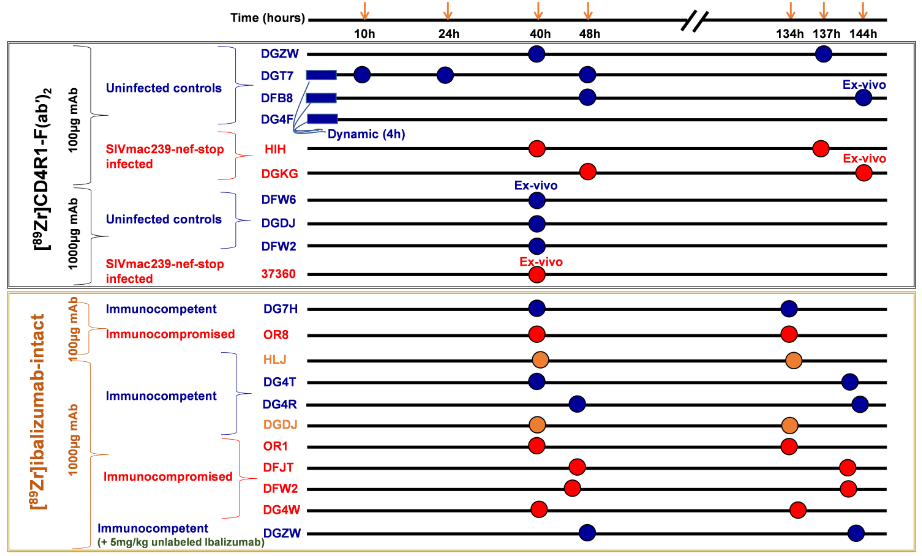


**Fig. S1** Imaging schema. Blue labels are healthy uninfected controls, Red labels are immunocompromised SIV-infected, and Orange labels are immunocompetent SIV-infected rhesus macaques.


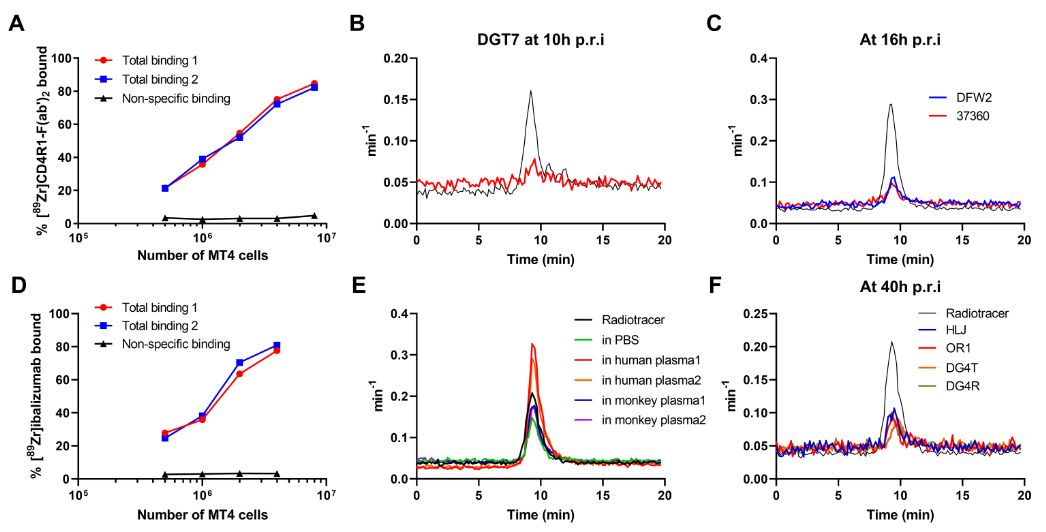


**Figure. S2** Total binding (duplicates: red and blue) and non-specific binding (with >3 log excess amount of the unlabeled mAb; black) of (**A**) [^89^Zr]CD4R1-F(ab′)_2_ and (**D**) [^89^Zr]ibalizumab to CD4 receptor was tested in-vitro using MT4 cells. [^89^Zr]CD4R1-F(ab′)_2_ (black) stability was tested *in-vivo* using size exclusion radio-HPLC analysis of plasma obtained at (**B**) 10h in DGT7 and (**C**) 16h in DFW2 (blue) and 37360 (red) post-radioligand injection. More than 90% of radioactivity of the injected plasma (red curve) eluted with a retention time identical to that of radiotracer (black curve), suggesting that the imaging probe was stable *in-vivo*. [^89^Zr]ibalizumab (black) stability was tested in (**E**) PBS (green), human plasma (red and orange), and rhesus macaque plasma (blue and purple) at 37°C at 40h and (**F**) *in-vivo* in plasma obtained at 40h post-radioligand injection. [^89^Zr]ibalizumab was stable at 37°C incubation for 40h in PBS, human, or monkey plasma. *In-vivo* plasma at 40h post-injection also showed that the imaging probe was stable *in-vivo* as more than 90% of the radioactivity was eluted at the retention time identical to that of the radiotracer. Radiochromatograms were transformed into probability density curves by normalizing for the area under the curve.


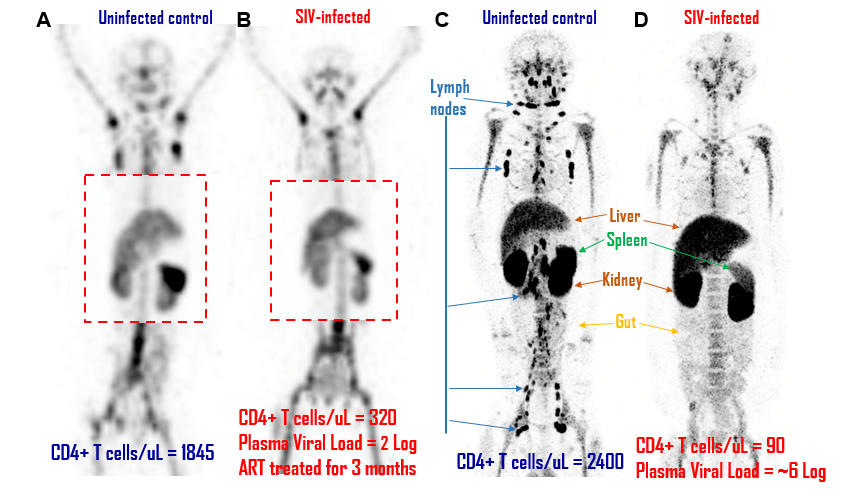


**Figure. S3** Maximum intensity projection *in-vivo* SPECT and PET images of rhesus macaques following administration of 100µg mass of CD4R1-F(ab′)_2_ radiolabeled with ^99m^Tc for SPECT and ^89^Zr for PET. SPECT images were obtained at 21h post-injection (**A**) of 152.4MBq and (**B**) 223.1MBq of ^99m^Tc-probe, and PET images at 40h post-injection (**C**) of 57.7MBq (in DGZW) and (**D**) 59.9MBq (in HIH) of ^89^Zr-probe. Due to higher partial volume effect in SPECT, two different thresholds were used to represent SPECT images (SUV=3 is overlapped with abdominal SUV=10), but only one threshold of SUV=15 was used for PET images. Note, better resolution of PET imaging (C, D) where individual vertebral body are visualized.





**Figure. S4** Comparison of mean standardized uptake value (SUVmean) in tissues and SUV in plasma between uninfected controls (blue) and SIV-infected animals (red), and between (**A**) 100µg (3 uninfected controls and 2 SIV-infected) and (**B**) 1000µg (3 uninfected controls and 1 SIV-infected) [^89^Zr]CD4R1-F(ab′)_2_ mass groups at 40-48h post-injection. Comparison of (**C**) maximum and (**D**) mean SUV in tissues and SUV in plasma between uninfected controls (blue) and SIV-infected animals (red) imaged with 100µg mass of [^89^Zr]CD4R1-F(ab′)_2_ at 40-48h (3 uninfected controls and 2 SIV-infected; solid) and 137-144h (2 uninfected controls and 2 SIV-infected; pattern) post-injection. Plasma SUV was calculated from the gamma counter. Plots are mean values and error bars are standard deviation.





**Figure. S5** (**A**) Maximum intensity projection *in-vivo* PET images of one uninfected rhesus macaque (animal ID: DGT7) following administration of 100µg mass of [^89^Zr]CD4R1-F(ab′)_2_ and scanned at 10h, 24h, and 48h post-injection. Tissue uptakes were converted to RAINBOW color map as shown in the color bar, where the red color indicates a high standardized uptake value (SUV). (**B**) maximum SUV (SUVmax) and (**C**) mean SUV (SUVmean) of radioligand uptake in various tissues over time.


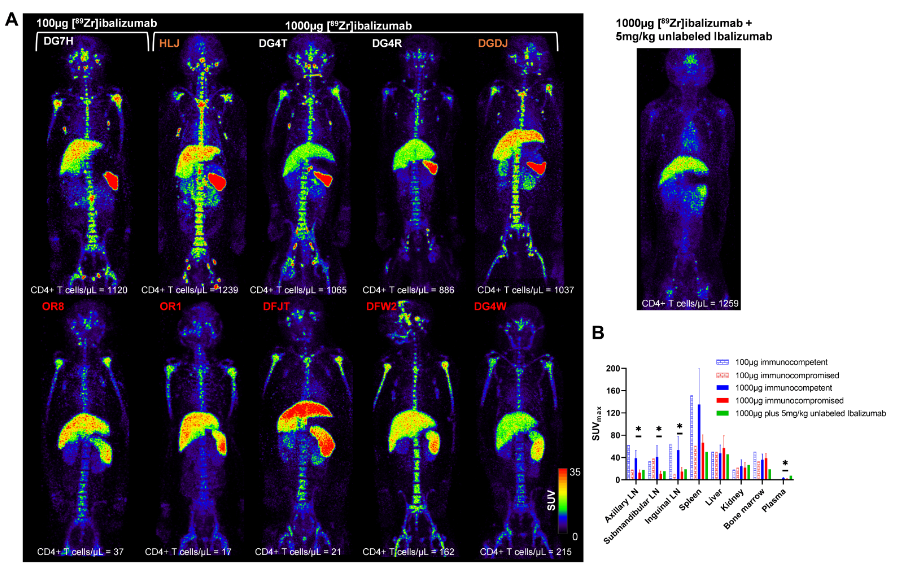


**Figure. S6 [^89^Zr]Ibalizumab biodistribution at 134-144h post-injection.** (**A**) Maximum intensity projection *in-vivo* PET images of rhesus macaques showing the comparison of radioligand uptake between immunocompetent (DG7H, HLJ, DG4T, DG4R, and DGDJ) and immunocompromised animals (OR8, OR1, DFJT, DFW2, and DG4W) following administration of 100µg (DG7H and OR8) or 1000µg mass (HLJ, DG4T, DG4R, DGDJ, OR1, DFJT, DFW2, and DG4W) of [^89^Zr]ibalizumab and scanned at 134-144h post-injection. Radiotracer specificity was demonstrated in one immunocompetent uninfected control (DGZW) by co-injecting 1000µg of [^89^Zr]ibalizumab with 5mg/kg of unlabeled ibalizumab. Tissue uptakes were converted to RAINBOW color map as shown in the color bar, where red color indicates the high standardized uptake value (SUV). (**B**) Comparison of maximum SUV (SUVmax) in tissues and SUV in the plasma between immunocompetent (100µg: blue, pattern, n = 1; 1000µg: blue, solid, n = 4), immunocompromised (100µg: red, pattern, n = 1; 1000µg: red, solid, n = 4), and CD4-blocked with excess unlabeled Ibalizumab animals (green, n = 1) at 40-48h post-injection. Plasma SUV was calculated from the gamma counter. Plots are mean values and error bars are standard deviation. Statistically significant differences in radiotracer uptakes (indicated by * for P-value < 0.05) were observed in lymph nodes and plasma between the immunocompetent and immunocompromised animals that received 1000µg of [^89^Zr]ibalizumab.


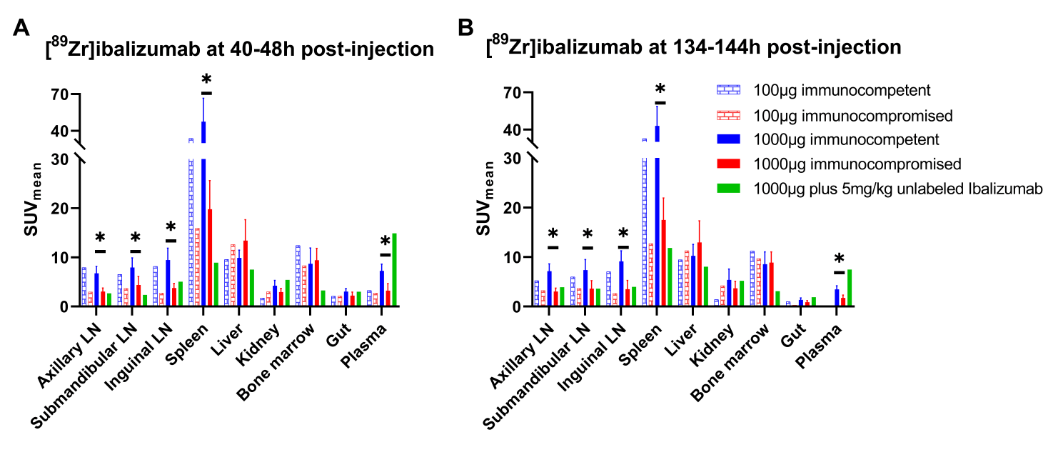


**Figure. S7** Comparison of mean SUV (SUVmean) in tissues and SUV in the plasma of [^89^Zr]ibalizumab between immunocompetent (100µg: blue, pattern, n = 1; 1000µg: blue, solid, n = 4), immunocompromised (100µg: red, pattern, n = 1; 1000µg: red, solid, n = 4), and CD4-blocked with excess unlabeled Ibalizumab animals (green, n = 1) at (**A**) 40-48h post-injection and (**B**) 134-144h post-injection. Plasma SUV was calculated from the gamma counter. Plots are mean values and error bars are standard deviation. Statistically significant differences in radiotracer uptakes (indicated by * for P-value < 0.05) were observed in lymph nodes, spleen, and plasma between the immunocompetent and immunocompromised animals that received 1000µg of [^89^Zr]ibalizumab.


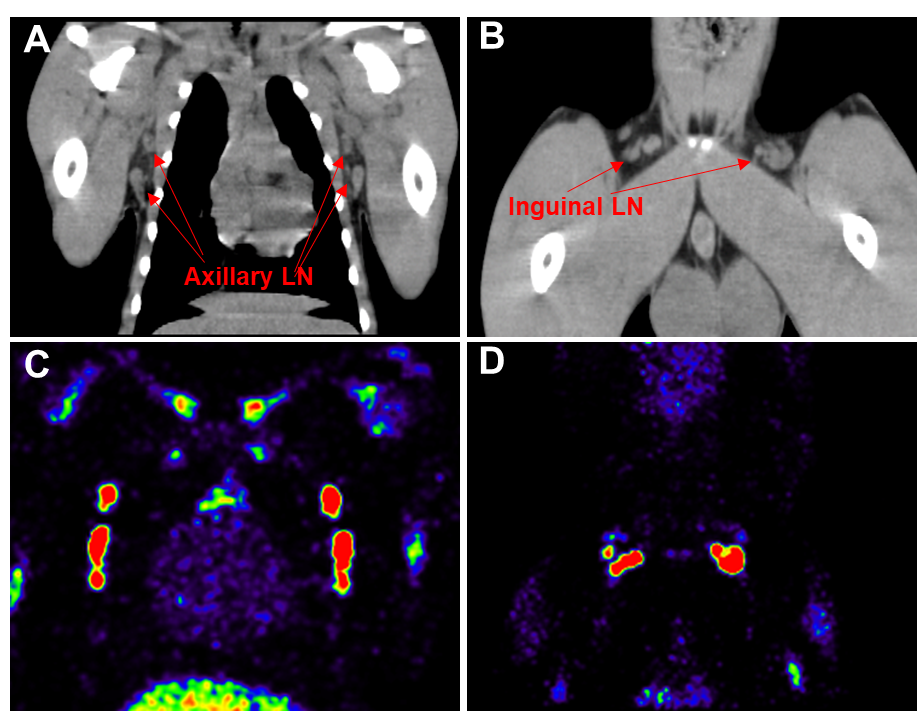


**Figure. S8** CT (top panels) and PET (bottom panels) coronal view of the clusters of axillary (panels A and C) and inguinal lymph nodes (panels B and D) in one uninfected rhesus macaque.





**Figure. S9** Time-activity data following an intravenous bolus injection of 100µg mass of [^89^Zr]CD4R1-F(ab′)_2_ in two uninfected controls - DFB8 (panel A) and DGT7 (panel B). Plots show the concentration of radioligand in arterial whole-blood (blue) and arterial plasma (red) from femoral bleeds, and the whole-blood activity derived from a volume of interest (VOI) placed over cardiac blood pool of PET image (green) during the 4-hour dynamic scan. The Area Under the Curve (AUC_0-240min_) of radioactivity concentration of the whole-blood from femoral arterial bleeds (DFB8: 392.8 and DGT7: 422.6 µCi*min/mL) overlapped well with the AUC_0-240min_ of the image-derived whole-blood radioactivity concentration (DFB8: 441.1 and DGT7: 483.9 µCi*min/mL).

**

**

**Figure. S10** (**A**) Three-tissue compartment model with metabolites. In addition to the two-tissue compartment model, a input curve (C_m_) of the metabolite enters the tissue and undergoes non-specific binding to form metabolized ligand in tissue (C_M_). (**B**) Three-tissue compartment model with internalization. After the specific binding of the ligand to the receptor, the ligand-receptor complex is internalized (C_I_).

**Movie S1** Maximum intensity projection *in-vivo* PET images of rhesus macaques following administration of 100µg mass of [^89^Zr]CD4R1-F(ab´)_2_. Video shows the comparison of radioligand uptake between an uninfected control (DGZW) and an SIV-infected animal (HIH) scanned at 40h post-injection.

**Movie S2** Maximum intensity projection *in-vivo* PET images of rhesus macaques following administration of 100µg mass of [^89^Zr]CD4R1-F(ab´)_2_. Video shows rapid initial changes of radioligand uptake in a 3-field-of-view dynamic scan for 4h post-injection in an uninfected control (DG4F). Tissue uptakes were converted to RAINBOW color map as shown in the color bar, where the red color indicates the high standardized uptake value (SUV).
